# Inherited Susceptibility to Cancer: Past, Present and Future

**DOI:** 10.1111/ahg.70013

**Published:** 2025-07-21

**Authors:** Shirley V. Hodgson, William D. Foulkes, Eamonn R Maher, Clare Turnbull

**Affiliations:** ^1^ Department of Clinical Genetics, St George's School of Health and Medical Sciences City St George's, University of London London UK; ^2^ Departments of Human Genetics, Medicine and Oncology McGill University Montreal Quebec Canada; ^3^ Aston Medical School Aston University Birmingham UK; ^4^ Department of Genomic Medicine University of Cambridge Clinical School Cambridge UK; ^5^ Division of Genetics and Epidemiology, Institute of Cancer Research London, Department of Cancer Genetics Royal Marsden NHS Foundation Trust London UK

## Abstract

Germline pathogenic variants (GPVs, ‘mutations’) causing inherited susceptibility to certain cancers (cancer susceptibility genes, CSGs) broadly belong to one of two main classes—loss of function variants in tumour suppressor genes (TSGs) or gain of function variants in proto‐oncogenes (an over‐simplification). Genomic analyses of tumours identify ‘driver mutations’ promoting tumour growth and somatic variants which contribute to ‘mutation signatures’ which, with histopathology, can be used to subclassify cancers with implications for causality and treatment. The identification of susceptible individuals is important, as they and their relatives may be at elevated risk of tumours, and this can influence optimal cancer treatment. Classically, cancer risk assessment utilises family history, lifestyle/environment factors, and any non‐neoplastic clinical findings, followed by genetic testing of high/moderate penetrance CSGs. In cancer cases not caused by highly penetrant CSGs, multiple variants conferring relatively small risks play a major role. These were discovered by genome‐wide association (GWAS) studies. The utility of polygenic risk scores (PRS) derived from multiple such variants for clinical risk profiling is being assessed. Access to genetic tests is improved by widening eligibility criteria for testing and empowering non‐genetic clinicians to identify CSG GPVs and manage carriers. This will contribute to expanding programmes of screening, prevention and early detection (SPED), with personalised surveillance and prophylactic interventions, and exploit knowledge of the molecular mechanisms of cancer susceptibility to develop novel cancer therapies. In some jurisdictions, population testing is being considered, but GPV penetrance in this setting can be unclear, and the public health implications are complex.

## 1 Brief Historical Review

Our understanding of clinical cancer genetics had a stuttering start, with the description of the occasional family in which several close relatives developed the same type of cancer. Harrison Cripps and separately Broca described familial cancer families in the nineteenth century, and Warthin described what he termed ‘cancerous fraternities’, suggesting there was ‘some influence of heredity on cancer’ (Campos et al. 2024). Henry Lynch delineated ‘Lynch syndrome’ (LS) as an autosomal dominant ‘hereditary non‐polyposis colorectal cancer syndrome’ on restudying ‘Family G’ (documented by Warthin 1985) with multiple cases of colorectal and uterine cancers (Douglas et al. 2005). This condition, now renamed LS, causes an increased risk of colorectal, uterine and, to a lesser extent, certain other cancers. Penrose et al. (1948) demonstrated an increased relative risk of breast cancer in the close relatives of cases, and ‘hereditary breast/ovarian cancer’ (HBOC) families were later recognised as hereditary breast and ovarian cancer (Lynch et al. 1994). Hereditary conditions with a characteristic clinical phenotype which also predispose to specific cancers were described, such as familial adenomatous polyposis (FAP) (Dukes 1952), Neurofibromatosis Type 2 (Ruggieri et al. 2018), and Neurofibromatosis Type 1 (Bizzari and Bottaro 2015).

## 2 Gene Identification and Function

The genes underlying cancer susceptibility were identified by a variety of techniques. The location of the *APC* gene, in which GPVs are responsible for FAP, was identified via the detection of a deletion in Chromosome 5 in individuals with FAP (Herrera et al. 1986; Bodmer et al. 1987), identifying this as the location of this tumour suppressor gene.

Linkage analysis was employed to identify the genes in which GPVs caused a strong predisposition to specific cancers, such as *BRCA1 and BRCA2* causing HBOC (Miki et al. 1994; Wooster et al. 1994, 1995), and the genes in which GPVs caused LS, *MLH1, MSH2, PMS2* and *MSH6* (Fishel et al. 1993; Lynch et al. 2009) (the ‘low hanging fruit’). Both conditions cause a significant lifetime risk of the relevant cancers and are due to specific DNA repair defects (defective homologous recombination DNA repair in HBOC, DNA mismatch repair defects in LS). HBOC is manifested by a significantly increased risk of breast and ovarian cancer at a younger age than in the general population, with the greatest contribution from GPVs in *BRCA1* and *BRCA2*. Subsequently, the candidate gene approach was used to identify moderately penetrant genes, where families segregating for multiple cases of the relevant tumours were less frequent, making linkage analysis more difficult. Candidate genes with similar functions to CSGs already identified were chosen, such as DNA repair defects, for example, *CHEK2*, a moderate‐risk breast cancer susceptibility gene which participates in the same DNA repair pathway (homologous recombination) as *BRCA1* and *BRCA2* (Meijers‐Heijboer et al. 2002).

Inherited defects in TSGs as causes of cancer susceptibility had been suggested by Knudson (1971) by documenting that bilateral (inherited type) retinoblastoma appeared to have a time‐to‐diagnosis curve consistent with a single acquired (somatic) rate‐limiting mutation, whereas in sporadic unilateral cases, the age‐at‐onset was consistent with two somatic rate‐limiting mutations (i.e., in both cases, two mutations/‘hits’ are required for tumour initiation). This led to the paradigm that inactivation of both copies (alleles) of a TSG is required to initiate carcinogenesis, with the inherited TSG PV present from conception in susceptible individuals, so it is only the second TSG allele that requires inactivation, by deletion, mutation or epigenetic modification such as methylation, to initiate the relevant tumour. Many cancer susceptibility conditions are caused by defective TSGs, including FAP, in which GPVs in the *APC* gene result in increased Wnt signalling via the accumulation of beta‐catenin, causing adenomatous polyposis with chromosome instability and aneuploidy. *APC* mutations are well established as driver mutations in colorectal cancer, and the APC protein is also involved in signalling pathways other than Wnt and is a cytoskeletal regulator (Abbott and Nathke 2023). The hamartomatous polyposis syndromes are caused by GPVs in the TSGs *SMAD4* and *BMPR1A*, which reduce regulation of TGF‐beta signalling. Such conditions are often associated with specific clinical phenotypes, such as melanotic freckling of some areas of the skin in Peutz–Jeghers syndrome, caused by GPVs in *STK11*, which compromises the control of cellular proliferation via G1 cell cycle arrest and apoptosis (Lim et al. 2003; Bresler et al. 2016; S. Hodgson 2008; Glaire et al. 2017).

Inherited GPVs causing constitutional upregulation of proto‐oncogenes can act as CSGs; for example, activating mutations in the *RET* oncogene cause multiple endocrine neoplasia type 2 (MEN2), a condition predisposing to medullary thyroid cancer and phaeochromocytoma (Eng 1996). Interestingly, different GPVs in this gene can cause different phenotypes: the milder MEN2A, some cases being associated with lichen amyloidosis or Hirschsprung's disease (with a lack of intestinal ganglia), or a more severe cancer phenotype, MEN2B, that can manifest as intestinal ganglioneuromatosis and mucosal neuromas (Brain et al. 2025).

Many CSGs are related to cell‐cycle regulation and DNA repair, as in HBOC and LS. LS is due to GPVs in genes involved in MMR post‐replicative DNA surveillance, causing a hypermutated state in colorectal polyps with increased adenoma to carcinoma progression (Lynch and de la Chapelle 2003). The most prevalent cause of HBOC is inherited GPVs in *BRCA1* and *BRCA2* and, more rarely, *TP53*, the most penetrant genes commonly involved, but lower penetrance genes also involved in the same DNA repair pathway can cause a similar phenotype, including *PALB2, RAD51C* and *RAD51D* (Rowlands et al. 2024). Defective DNA repair mechanisms are also prominent in CSGs, causing colorectal cancer susceptibility. Biallelic GPVs in *MUTYH*, involved in the repair of oxidative DNA damage, cause an autosomal recessive adenomatous polyposis resembling FAP. This was initially deduced from the fact that colonic neoplasia in this condition displayed an excess of G:C > T:A transversions, as would be expected if there was defective DNA base‐excision repair (after oxidative damage) (PMID: 11818965; Tomlinson 2015). A further type of DNA repair defect, due to inherited GPVs in *POLD1* and *POLE*, DNA polymerase proofreading genes, which normally repair DNA damage due to incorrectly inserted nucleotides in DNA, results in an autosomal dominant susceptibility to endometrial cancer and colonic polyps, with a hypermutator phenotype in the neoplastic lesions (Palles et al. 2013). This induces an increased immune response, which may be related to an improved prognosis in endometrial cancers bearing such mutations. Inherited *NTHL1* GPVs cause an autosomal recessive susceptibility to multiple cancers, also due to a DNA base excision repair defect (Rivera et al. 2015; Weren et al. 2015). There is also an autosomal recessive multi‐tumour predisposition syndrome (with colorectal adenomas, acute myeloid leukaemia and uveal melanoma) associated with biallelic germline mutations in *MBD4*, a gene which cooperates with *MUTYH* in oxidative damage repair (Palles et al. 2022).

Other CSGs are involved in angiogenesis, where the pathogenic mutations cause overactivity of the vascular endothelial growth factor (VEGF) and other hypoxia‐inducing factor (HIF) target genes, as in von Hippel–Lindau disease (VHL), causing an increased risk of vascular tumours such as renal cell carcinoma and cerebellar hemangioblastoma (Maher et al. 2011).

Most cancer susceptibility conditions appear to be inherited as autosomal dominant traits, but it is more difficult to reliably identify late‐onset recessive traits when sibships are small in most populations where we have access to detailed cancer history data. A few cancer susceptibility traits have known autosomal recessive inheritance, such as polyposis caused by inherited GPVs in *MUTYH* and *MBD4* (see above). Occasionally, childhood‐onset cancer may occur in individuals with biallelic GPVs in conditions where adult‐onset cancers are characteristic in individuals heterozygous for the GPV, as with *BRCA2* (causing Fanconi anaemia, *FANCD1* in biallelic GPV carriers), although homozygous GPVs in such genes (particularly if truncating, or complete loss‐of‐function) may result in embryonic lethality (Rahman and Scott 2007). Individuals with biallelic GPVs in LS genes (usually GPVs of low penetrance for LS in heterozygotes) cause a condition known as constitutional mismatch repair deficiency (CMMRD). This is a rare disorder causing a susceptibility to a variety of cancers in early childhood, including colorectal and brain cancers and leukaemia (Bakry et al. 2014).

A few conditions due to GPVs in TSGs demonstrate parent‐of‐origin transmission. Thus, maternally inherited loss‐of‐function mutations in *CDKN1C*, a maternally expressed imprinted gene, are associated with the Beckwith–Wiedemann congenital overgrowth syndrome (Lam et al. 1999), which is associated with an increased risk of Wilms tumour, hepatoblastoma, neuroblastoma, rhabdomyosarcoma and other embryonal cancers. Interestingly, with inherited *SDHD* GPVs, where (almost) only paternal transmission of the GPV causes paraganglioma susceptibility in the offspring, *SDHD* is apparently not an imprinted gene (Burnichon et al. 2017).

Genes involved in cell senescence have been implicated in melanoma susceptibility (Constantinou and Bennett 2024). Inherited alterations in the *DICER1* gene can cause an autosomal dominant genetic susceptibility to several tumour types, especially childhood‐onset pleuropulmonary blastoma (PPB) and other tumours including those of the kidney, thyroid, ovary, cervix, brain and eye. This gene encodes a protein which is part of the micro‐RNA (miRNA) biogenesis pathway, regulating the activity of other genes via the production of miRNAs, short non‐coding RNA molecules which reduce the expression of mRNAs that they target via sequences in their 3′ gene untranslated regions (UTRs) (Foulkes et al. 2014). Important targets include mRNAs encoding oncofoetal proteins (Fraire et al. 2024). Other members of the miRNA biogenesis pathway are also implicated in tumorigenesis (Pelletier et al. 2023). Registries for individuals with this rare condition have been developed, improving the delineation of this condition (Schultz et al. 2023).

The functions of CSGs are often ubiquitous, raising the question of why inherited defects in such universally important gene functions can result in susceptibility to a restricted number of cancers and often of specific types of cancer, for example, triple‐negative breast cancers in *BRCA1* heterozygotes (Rahman 2014b).

The penetrance of GPVs causing inherited cancer susceptibility can vary with different mutations in the same gene, which can cause altered degrees of cancer susceptibility and tumour spectrum and even different clinical phenotypes, for example, as noted above with *RET* GPVs in MEN2 and mutations in *TGFBR1*, where missense mutations can cause a Marfanoid‐like vasculopathy or Loeys–Dietz syndrome, but others (e.g., truncations or missense mutations in specific domains of the gene) can cause Ferguson–Smith syndrome (self‐healing squamous epithelioma) (Goudie et al. 2011). In some cases, the type of mutation in the causative gene can give clues as to the severity of the phenotype, as in FAP, where certain GPVs in the *APC* gene cause characteristic phenotypic expression such as attenuated phenotype, expression of desmoids or retinal lesions known as CHRPE, or risk of gastric cancer (Friedl and Aretz 2005).

Cancer susceptibility genes probably only account for about 5%–10% of inherited cancers. Genetic variants with low penetrance (SNPs) that individually alter cancer susceptibility to a small extent were detected by genome‐wide association studies (GWAS), but since on their own they have only minor effects on cancer risks, they are not used individually for stratifying risk in clinical practice. However, multiple SNPs can be utilised to develop a polygenic risk score (PRS), and risk‐associated SNPs can also give some insights into the mechanisms involved in susceptibility to specific cancers, such as the recent identification of multiple SNPs associated with colorectal cancer in large GWAS studies (Fernandez‐Rozadilla et al. 2023; Zhang et al. 2021). Concerns regarding the use of such variants for disease risk estimates include the need for multiple variants to be tested and the weaker evidence for their utility in non‐European individuals because GWAS analyses have to date mostly been done in European cohorts (Lewis and Vassos 2020, Yang et al. 2024). However, since most conditions are due to multiple variants in low‐penetrance genes, it has been proposed that a combination of selected variants might be used with other genetic and lifestyle/environmental data to derive a more accurate estimate of an individual's cancer risk. Increasing the size of the cohort used for the GWAS increases the predictive value of the PRS to the limit of the trait heritability (Ndong Sima et al. 2024).

## 3 Clinical Cancer Genetics

Consultant‐led clinical cancer genetics services were set up in the US, Europe and Canada in the 1970s–80s (S. V. Hodgson et al. 2014). Nurses and genetic counsellors were later added to the clinical services, with suitable training protocols (Gulzar et al. 2007). Multidisciplinary clinics were also set up. Pre‐clinic assessment of family history was provided at clinics with appropriate manpower, and digital input of data is now being developed in some centres to facilitate patient triage (Youngs et al. 2024).

Cancer risk assessment and management are increasingly managed outside of a specialist genetic clinical setting, such as in a breast unit or colorectal surgical clinic. However, assessment of the pathogenicity of novel GPVs is best addressed in consultation with the clinical genetics team. As mainstreaming of genetics services is being increasingly advocated, devolving straightforward cases to the relevant non‐genetic specialities, close collaboration with the genetics services should be promoted, possibly using a dedicated specialist genetics nurse in such clinics (Monaghan et al. 2024; MacFarland et al. 2024), to help interpret some genetic test results and arrange cascade testing and management of the extended families of individuals with GPVs in CSGs (Bennett et al. 2010; al Bakir et al. 2019; Hallowell et al. 2019). Currently, many non‐geneticists feel ill‐equipped to conduct genomic medicine, so improved genetics education is being developed (Weren et al. 2015).

## 4 Maximising the Identification of Individuals With an Inherited Cancer Predisposition Disorder

To reduce morbidity and mortality from inherited cancer predisposition, it is necessary to (a) maximise the identification of at‐risk individuals, (b) then apply cost‐effective early detection or preventative strategies to those at increased risk, and (c) develop effective targeted therapies for when cancer does occur in individuals with GPVs in a CSG.

Traditionally, at‐risk individuals were identified because a clinical or genetic diagnosis of an inherited cancer syndrome was made following a cancer diagnosis or because their relative was diagnosed with an inherited cancer syndrome. Prior to CSG identification, a precise diagnosis of an inherited cancer syndrome relied on the presence of distinctive clinical or pathological features and was limited to a restricted number of diagnoses (e.g., extensive polyposis in FAP) (Friedl and Aretz 2005) or combinations of rare tumour types in VHL disease, MEN2, BHD syndrome and so forth (Hodgson 2008). For non‐syndromic disorders such as LS, clinical diagnostic criteria that relied on a threshold number of affected individuals, age at diagnosis and the familial relationships between affected individuals were adopted (e.g., Amsterdam criteria for LS) and revised as knowledge of the disorder increased (Beck et al. 1997). When a clinical diagnosis was made, affected and at‐risk family members were then offered relevant genetic counselling and surveillance/preventative interventions (e.g., thyroidectomy in MEN2). The identification of the genes responsible for familial/bilateral retinoblastoma, NF2, VHL disease, FAP, LS, HBOC, and so forth, enabled the molecular confirmation of a clinical diagnosis and presymptomatic diagnostic testing for at‐risk relatives (S. V. Hodgson et al. 2014). For inherited cancer disorders, a major benefit of the latter is that relatives who test negative for the familial GPV can be reassured and excluded from surveillance programmes.

**FIGURE 1:**
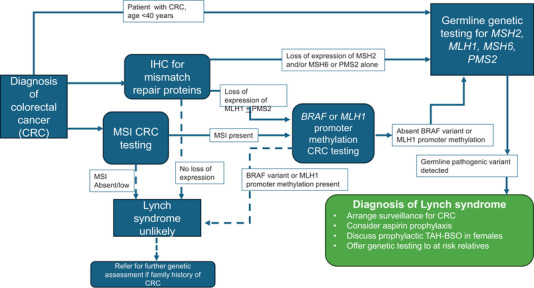
Pathways for investigation of individuals with colorectal cancer (CRC) to identify those with Lynch syndrome (LS) and clinical interventions in people with a diagnosis of LS. IHC, immunohistochemistry; MSI, microsatellite instability; TAHBSO, total abdominal hysterectomy and bilateral salpingooophorectomy (figure adapted from Edwards and Monahan 2022).

Screening for LS can be done by testing colorectal tumour samples for microsatellite instability or abnormal immunohistochemistry, indicative of LS (Hampel et al. 2008). In order to maximise the ascertainment of individuals with LS, genetic counselling and testing are advised for individuals diagnosed with colorectal cancer under the age of 40 years. All persons with colorectal or endometrial cancer should have their tumours tested for microsatellite instability or immunohistochemistry for mismatch repair proteins. Those demonstrating abnormalities of MLH1 (or MLH1 and PMS2) are further tested for MLH1 promoter methylation and, in the case of CRC, for the BRAF p.V600E mutation using the algorithm illustrated in Figure 1, where individuals whose tumours show MLH1 promoter hypermethylation or BRAF mutations are unlikely to have LS; those without, and all cases showing loss of other MMR proteins, or loss of combinations of MMR proteins, are recommended to have testing for germline mutations in the LS genes. (Figure 1) (Edwards and Monahan 2022).

Widening the criteria for genetic testing of tumours or individuals with cancer can increase the detection rate of CSGs but requires a public health assessment of the cost‐benefit (Snowsill et al. 2014). How results and their implications are to be given to patients and their doctors also requires consideration (Rahman 2014a).

In many inherited cancer predisposition disorders, the availability of molecular diagnostic testing revealed that many (in some cases most) individuals with a GPV did not satisfy clinical diagnostic criteria for the relevant disorder and that clinical diagnostic criteria and/or molecular diagnostic testing eligibility criteria required revision to reduce the number of undiagnosed persons with a GPV. To improve genetic testing in familial breast cancer, a clinical scoring system (‘Manchester score’) was developed to assess the likelihood of a *BRCA1* or *BRCA2* GPV variant being present in a family based on the presence of pathological type, age at onset and number of breast and other cancers in close relatives with a threshold score at which genetic testing was recommended (G. R. Evans and Lalloo 2010; D. G. Evans et al. 2017). More recently, more complex computer algorithms such as CanRisk have been developed which incorporate, in a Bayesian fashion, clinical features, family history, imaging data, lifestyle factors, hormonal and reproductive history and the results of genetic analysis to provide personalised breast and ovarian cancer risks and, in GPV negative cases, risks of an underlying genetic cause (Walker et al. 2015; Kalia et al. 2024; Tsoulaki et al. 2024). We anticipate that this comprehensive risk analysis approach to inform testing, surveillance and management decisions will be extended to all tumour types with the increasing application of machine learning/AI studies of genetic predisposition to cancer.

There may be perceived concerns by individuals taking a genetic test that a positive test indicating an increased cancer risk may cause problems with employability or the ability to obtain health insurance. This has been mitigated in the UK by an agreement between the Government and the Association of British Insurers (the Code on Genetic Testing and Insurance), which means that a company that has signed up to the code is required not to request or pressurise someone to take a genetic test and not to request or take into account the results of a genetic test in insurability decisions.

Individuals at increased risk of specific cancers because of young age at diagnosis or family history of the relevant cancers may be offered panel tests incorporating the genes most often involved in causing such susceptibility, such as a panel including *BRCA1, BRCA2, PALB2, RAD51C, RAD51D, PTEN, CDH1* and *TP53* for individuals with possible HBOC (Bouras et al. 2023).

As the cost of DNA sequencing falls, it may become feasible to test most or all affected individuals with specific tumour types. Approximately 40% of all individuals with phaeochromocytoma or paraganglioma have a GPV, and under the current eligibility criteria for genetic testing in the UK, most affected patients will qualify for testing (Lussey‐Lepoutre et al. 2024). Germline genetic testing may be performed when universal tumour sequencing is instigated for specific tumour types. Since 2023, the NHS has offered whole genome sequencing to all children with cancer. This may facilitate more accurate diagnosis, the use of targeted therapies (when available) and the identification of those with an inherited cause (Hodder et al. 2024).

Ethical considerations are important in planning such developments, and adequate informed consent and the possibility of unintended secondary findings need to be considered carefully in developing such clinical pathways.

Issues that need to be considered include the inadvertent identification of a condition conferring cancer susceptibility during a genomic test of a child or newborn. The usual practice when offering genetic testing for cancer susceptibility in a familial setting is not to offer predictive tests for a known familial mutation in childhood unless a positive test will result in surveillance or other prophylactic measures in childhood. If such a CSG GPV is inadvertently detected in a newborn or childhood genomic test for a different indication, the result needs careful evaluation by a clinical multidisciplinary team, considering how and whether to disclose the result. Ideally, the possibility that such an unexpected test result could be obtained should be discussed with the parents prior to the test.

With the increasing application of large‐scale genome sequencing initiatives for research and healthcare, there is an ever‐increasing number of rare genetic variants being discovered, and if these are identified in individuals with possibly heritable conditions, particularly if they occur in a potentially relevant candidate gene, it may be unclear whether the variant is pathogenic or not. Such variants of uncertain significance (VUS) are problematic for clinical management decisions, so there have been increasing numbers of initiatives to facilitate the resolution of VUSs to benign or pathogenic classes. VUSs are more likely to occur when there is limited information on genetic variation in a healthy population, for example, in communities/ethnic groups that originate outside of regions, such as Europe and North America, in which clinical genomics is in routine use. Large compilations of genomic databases such as gnomAD and UK Biobank are important sources of data for variant interpretation, especially when supplemented with data from studies such as Genes&Health (Bodian et al. 2014), which focuses on ethnic groups not well represented in gnomAD and UK Biobank (Allen et al. 2024). Data sharing through ClinVar, Decipher and similar initiatives, together with more standardised processes for classifying variant pathogenicity under the ACMG‐AMP variant interpretation guidelines, are also important for facilitating accurate variant interpretation. Accurate delineation of the effects of VUSs on the function of the relevant gene product is valuable but, until recently, has been available for relatively few genes/variants. The recent availability of MAVE (Multiplexed Assays of Variant Effects) studies provides a powerful tool to enable a comprehensive high‐throughput approach to systematically evaluate variant‐function relationships for CSGs such as *BRCA1/BRCA2, VHL*, and so forth (Fowler et al. 2023; Weile and Roth 2018). Recommendations are being drawn up to help assess the penetrance of VUSs (Schmidt et al. 2024).

The penetrance of GPVs in CSGs has in the past been assessed in families ascertained because of a strong family history of the condition. In contrast, the penetrance of the same GPVs ascertained in population series appears to be lower, often significantly lower. This effect may depend on which gene is involved and may be greater with some genes than others. When incidental GPVs of low penetrance are detected, it is difficult to decide on their actionability.

## 5 Management of Inherited Cancer Susceptibility Disorders

Individuals shown to be predisposed to the development of specific tumours may be managed by a variety of strategies, including surveillance and early detection and interventions to reduce cancer risks, for example, by removing the target organ or chemoprophylaxis.

In the case of VHL disease, the major organs at risk of tumour involvement (kidneys, cerebellum and spinal cord, retina, pancreas) are not appropriate for risk‐reducing preventive surgery; therefore, clinical management has focused on surveillance of the target organs and early detection of tumours. Comprehensive renal screening programmes demonstrated that very small tumours do not require immediate removal, and surgery can be postponed until they grow to a predefined diameter, whilst retinal lesions are mostly treated as soon as they are detected (Maher et al. 2011). Surveillance programmes for VHL disease are associated with improved patient survival (Wilding et al. 2012). Tumours in patients with VHL disease are primarily driven by the hypoxia‐inducible factor‐2 (HIF‐2) transcription factor, and the introduction of the small molecular HIF‐2 antagonist belzutifan offers a medical therapy for cases in which surgery is undesirable/unsuitable (Jonasch et al. 2021). Longer‐term, it is hoped that medical options to prevent or arrest tumour development at an early stage will be developed. Cascade testing for the close relatives of affected individuals found to be mutation carriers then opens up the possibilities of primary prevention and/or early detection strategies to reduce or prevent advanced cancers in these groups. In disorders such as MEN2, the risk of thyroid cancer can be prevented by prophylactic thyroidectomy. Women at high risk of breast and ovarian cancer, such as *BRCA1/BRCA2* heterozygotes, may choose prophylactic mastectomy and/or oophorectomy. Risk‐reducing salpingectomy is being assessed as a method of reducing the risk of ovarian cancer in women who carry a GPV in *BRCA1* or *BRCA2* before they are ready to consider oophorectomy (PROTECTOR Study, Sideris et al. 2024).

A colonoscopy every 2 years is usually offered to individuals with LS from an age determined by the gene involved. Individuals at a (lower) two‐to‐four‐fold colorectal cancer risk may be offered five yearly colonoscopies from 45 years, but recent studies suggest that a larger interval (up to 10 years) between colonoscopies can be sufficient for cancer prevention if 2‐yearly FIT tests (tests for blood in the stools) are added between colonoscopies (van Wifferen et al. 2025), reducing costs.

For most individuals at risk of an inherited cancer, surveillance programmes are the most frequent intervention to enable early cancer detection and reduce morbidity/mortality. The increased cancer risk in predisposed individuals can make surveillance modalities that would not be cost‐effective in a general population clinically effective and financially efficient in a high‐risk cohort (Thompson et al. 2023). However, the rarity of some inherited cancer syndromes can make it challenging to demonstrate the efficacy of surveillance in reducing mortality. Many surveillance programmes are based on imaging (e.g., MRI), and cost‐effectiveness could be improved if biomarkers (e.g., circulating tumour DNA) or PRS are used to stratify risk and focus the most intense screening on those most likely to develop a tumour. Research studies are currently underway to evaluate screening by liquid biopsy for early cancers. An infrastructure to track at‐risk individuals and ensure availability of screening for them on a regional/national scale is being developed and will be an important longer‐term development. Comprehensive registers for conditions such as LS are being set up to optimise the ascertainment of at‐risk family members and adherence to surveillance protocols (developed by national and international clinical cancer genetics groups such as the Cancer Genetics Group [CGG] in the UK). Hand‐held devices which alert patients when their next screening event is due are being trialled in Lynch and other syndromes with success. As an example, an LS App has recently been developed as a patient‐facing self‐navigation guide partnership between the East Genomics GMSA, the University of Leicester, Instant Access Medical, Lynch Syndrome UK and Day One Strategy. This uses CGG guidelines to assist patients in managing their condition. It provides a clinical management dashboard based on a coding system to guide patient care and potentially act as an anonymous audit of care.

Chemoprophylaxis to prevent the development of cancer/pre‐cancerous lesions (e.g., colorectal polyps) is an active area of interest. In LS, oral aspirin continuing for at least 2 years has been associated with a reduction in colorectal cancer risk and other relevant cancers (e.g., endometrial) (Serrano et al. 2022). The CAPP study found that a daily dose of 600 mg aspirin significantly reduced the risk of colorectal and other cancers in individuals with LS (Burn et al. 2020). This trial also reported a dietary fibre intervention which was successful (Mathers et al. 2022).

Currently, studies are underway to evaluate the role of metformin in preventing cancers in individuals with Li Fraumeni syndrome (caused by germline TP53 mutations) (Dixon‐Zegeye et al. 2024).

## 6 Targeted Therapies for Cancer Treatment

Genetic testing of tumours identifies specific mutational profiles which can betray the processes which have driven cancer development and may provide clues as to characteristics of the patient host, such as their age, mutagenic exposures (sunlight, smoking), or their ability to repair DNA defects. This, in addition to conventional histopathological data, can indicate the exact tumour subtype and derive specific targeted treatments (Cornish et al. 2024; Alexandrov et al. 2013). Personalised treatment of cancer can be facilitated using artificial intelligence (AI) (Abida et al. 2024).

Genetic testing of tumours can detect expressed tumour antigens for consideration for antibody treatment or mRNA vaccine development, possible immune therapy, or treatment related to a germline defect. The DNA repair defect in LS results in a failure of mismatch DNA repair, resulting in mutations in genes with short repeat sequences, such as *TGFBR2, BAX, MSH3* and *MSH6*. It is thought that these mutations result in rapid growth from early‐stage adenomas to carcinoma and cause an increased immunological response to the neopeptides in the cancers, as demonstrated by increased infiltration by lymphocytes via lymphovascular invasion. The development of cancer vaccines to target colorectal polyps and other early‐stage neoplastic lesions predicated on the presence of these immunogenic frameshift peptides is an area of great interest in LS as a possible preventive strategy (Gebert et al. 2021). Immune therapy in cancers in LS can also involve autologous T cell transfer or the use of modified T cells (Bowen et al. 2024).

Targeted treatments being developed include monoclonal antibodies (MAbs) against tumour growth factors, hormonal therapies such as tamoxifen to reduce growth‐promoting signals in breast cancers with oestrogen receptors, inhibitors of cell signalling from growth factors in the tumour, inducers of apoptosis, inhibitors of angiogenesis, immunotherapies, and toxic MAbs. PARP (Poly (ADP‐ribose) polymerase) inhibitors are effective for treating cancers in individuals carrying *BRCA1* mutations or women with triple‐negative cancers. This is predicated on the fact that *BRCA1*‐deficient cells are unable to perform DNA repair by homologous recombination, and PARP inhibitors prevent a different type of DNA repair, base‐excision repair, compromising the DNA repair ability of such tumours.

## 7 Recent Innovations in Screening and Prophylaxis in Cancer Susceptibility

Early detection of cancer in individuals with Li Fraumeni syndrome by ctDNA (shed by cancers) detected in blood is being evaluated as a biomarker of early cancers. The detection of ctDNA is also being evaluated as a screening test for lung cancer (Gale et al. 2022). Epigenetic alterations are thought to be involved in the carcinogenesis process, and the detection of aberrant DNA methylation patterns detected in circulating tumour DNA is being evaluated for the detection of early cancer and treatment responses (Geissler et al. 2024). A test (WID‐qEC) of methylation of the DNA of two genes (*ZSCAN12* and *GYPC*) performed on a cervicovaginal sample is being assessed for the detection of endometrial cancer in women with LS and *BRCA1/2* heterozygotes. This test can be self‐administered (Schreiberhuber et al. 2024).

In a new study, ‘MUSICaL’, microsatellite instability detected in DNA in urine samples is being assessed for the diagnosis of urothelial cancers in LS (Phelps et al. 2022).

Gene therapy using CRISPR‐Cas9 and related strategies is being developed for use in cancer treatment, such as producing modified T cells programmed to kill tumour cells in patients with refractory cancer, but this is in its early stages and requires careful regulation (Stadtmauer et al. 2020).

## 8 Heritability and Environmental Influences on Cancer Susceptibility

Twin studies comparing the concordance of different cancer types in monozygotic and dizygotic twins give an estimate of the heritability of these cancers, with an estimated heritability of cancer overall in one study being 33% (Mucci et al. 2016). Recent large‐scale twin studies have provided greater refinement regarding the proportion of cancer aetiology attributable to heritable (genetic) factors versus non‐heritable (environmental) factors: heritability estimates vary widely between cancer types, with, for example, the heritability of colorectal cancer estimated at 15% (0%–45%) compared to 57% (51%–63%) for prostate cancer (Mucci et al. 2016).

Large population studies are providing new opportunities to correlate genomic data with health and lifestyle data over time. The UK Biobank, for instance, was set up in 2006 and contains de‐identified genetic and health information from half a million participants aged 40–69 years, living in the UK. Regular repeat samples of blood, urine and saliva, and updates on health and lifestyle have been obtained, along with more detailed imaging in sizeable subsets. This aims to determine the influence of genomic factors and environmental influences on disease. A newer study, Our Future Health, aims to enrol 5 million adult volunteers, obtain genomic and health/lifestyle data on them, and derive disease risk scores with the aim of identifying individuals at increased risk of specific diseases. Providing surveillance for those at increased risk is expected to provide the opportunity to assess preventative and screening interventions.

In terms of non‐genetic factors, research is beginning to indicate that the microbiome in the gut and airways has an important role in influencing cancer susceptibility through a variety of mechanisms, including the inflammatory response. The discovery that stomach infection with *Helicobacter pylori* predisposes to gastric ulcers, adenocarcinoma and lymphoma gave clear evidence of the importance of the gut microbiome in cancer susceptibility (Mannion et al. 2021). Further research should provide deeper insights into these mechanisms and potential prophylactic and therapeutic interventions to reduce cancer risk (Jotshi et al. 2023). However, it is often difficult to establish a clear cause‐effect relationship between microbiome characteristics and disease risk. Exposition of the nonheritable component of cancer aetiology via epidemiologic studies remains slow: factors such as exercise, obesity and air pollution have been demonstrated, but most of the non‐genetic risk of cancers remains unexplained.

The rapid development of AI has profound implications for the improved understanding of genotype‐phenotype associations, predictions of protein structure and binding, and gene regulation. It is hard to predict how this will impact the future of cancer genetics, but it is likely to be very important.

## 9 Population Health Care Perspectives

Technology evolution over the last quarter century has afforded a wealth of discovery, with transformative sequencing methodologies facilitating identification of new cancer susceptibility genes, whilst SNP array technologies have enabled exposition of thousands of cancer‐associated common genomic variants combinable into polygenic scores. The concomitant adoption of these technologies by clinical diagnostic and commercial laboratories has transformed the scale, speed and cost of genomic analyses deliverable to patients and the public.

Previously, cancer genetics clinics featured multi‐case families affected with extreme, young‐onset and/or unusual cancer phenotypes, in whom testing of selected relevant gene(s) offered an aetiological explanation and perhaps a dichotomous prediction for the future for as‐yet unaffected family members. Genes were analysed sequentially via available low‐throughput technologies, with results often taking years to return.

We are seeing increasing momentum for cancer susceptibility genetic testing to move upstream of cancer patients (and their families) and to be offered to the general (well) public. This is reflected by the increasing policy focus on early detection and prevention of cancer in many healthcare settings. There was also a notable recent policy shift from the National Screening Committee in 2022 to move from strictly population‐based screening to encompass targeted and risk‐stratified screening. An example of this commitment is exemplified by the awarding of a substantial grant to the lead of the UK Cancer Data Driven Detection programme (Antonis Antoniou), with the aim of accessing and linking data from health records, genomics, family history, demographics, and behavioural data to develop advanced statistical models to predict those at increased risk of cancer. New AI tools will be developed to analyse this data to calculate an individual's lifetime risk of cancer.

There has been a sizeable UKRI and NHS funding commitment for national genomics infrastructure and programmes, including piloting of whole genome sequencing at birth with a view to a life‐course delivery model of timed ‘packages of genomic information’ to inform disease screening. In parallel, and in part absorbing displaced commercial post‐COVID‐19 capacity, there has been a recent surge in direct‐to‐consumer testing, including cancer screening tests, polygenic risk scoring and testing of cancer susceptibility genes. Whilst genetic risk stratification at the population level is feasible for many diseases, cancer is perceived to be a logical early use case, due in part to services already in place for screening and prevention. National cancer screening programmes exist for breast and colorectal cancer: there is enthusiasm that, via risk‐stratified allocation, these programmes might be ‘rationalised’ to improve outcomes and cost‐efficiency. There also exists a multitude of other interventions which have evolved from clinical genetics for the management of family members at elevated genetic risk of different cancers. Risk‐reducing bilateral salpingo‐oophorectomy stands out as a transformative intervention for women at elevated risk of tubo‐ovarian cancer (for which outcomes are otherwise poor); this offers significant benefit in ovarian‐cancer‐specific mortality with improvement in all‐cause mortality demonstrated in some studies. By contrast, the surveillance regimens we have developed for individuals ascribed as being of high genetic risk, for example, annual whole‐body MRI for musculo‐skeletal cancers and abdominal imaging for pancreatic cancers, have only been evaluated through small single‐arm studies of heterogeneous patient groups. As such, it is impossible to infer whether such a regimen offers any improvement in cancer‐specific mortality or whether perceived early diagnosis is just lead time. Overdiagnosis (and overtreatment) of indolent disease is also a major concern, particularly with multi‐modal, multi‐organ surveillance regimens.

As cancer susceptibility genomic testing moves from a low‐volume activity performed by a niche clinical speciality in unusual syndromic families, to being a diagnostic assay performed as routine in mainstream cancer clinics to a population‐level offering to the (largely) well public for stratification of screening, the opportunity for benefit (or harm) likewise scales up exponentially. Most medical tests examine for the presence/absence of disease today; by contrast, a positive genomic test result informs on long‐term (life‐long) risk, with accordant implications for decades of follow‐up, screening, interventions and potential psychological morbidity. Furthermore, unlike most medical tests, identification of a GPV in the index individual has implications for multiple family members. Scaled over time and ‘familial’ space, population‐level genomic testing thus has public health implications of considerable scale in regard to the consequent volume of downstream interventions for surveillance and risk management. Critical to the public health utility of cancer susceptibility gene testing are accurate estimates of penetrance for cancer, correct assignation of variant pathogenicity, the gene‐specific profile of cancer subtype and age‐specific association. Similar principles apply around the disease and disease sub‐type predictiveness and ancestry performance for polygenic scores. Also critical to the evaluation of the public health utility of any genetic test is the clinical efficacy of the interventions implemented for those at elevated risk. Has the surveillance intervention been proven to improve cancer‐related mortality? If so, do the mortality benefits exceed the harms?

It is important that we apply equivalent standards of public health scrutiny to proposed new models for population genomic screening (and the consequent downstream interventions) as those applied for other population health interventions and screening proposals. The extent to which we realise the benefits (and minimise the harms) from population‐level cancer genomic testing will be determined by the robustness with which we develop this infrastructure, expertise and regulatory standards in public health genomics.

Thus, to conclude, over the last 30 years, we have seen how the emphasis in clinically relevant cancer genetics has moved from discovery (1990–2005) to characterisation (2005–2020) and now to implementation. The true clinical value of the application of these extraordinary leaps in knowledge will need to be rigorously assessed to make sure the benefits outweigh the harms and access is equitable.

## Author Contributions

All the authors have made substantial contribution to the conceptualisation and design, planning and manuscript preparation or its critical revision of manuscript for intellectual content.

## Conflicts of Interest

The authors declare no conflicts of interest.

## Data Availability

The data that support the findings of this study are available on request from the corresponding author. The data are not publicly available due to privacy or ethical restrictions.
